# Adaptive false memory: Imagining future scenarios increases false memories in the DRM paradigm

**DOI:** 10.3758/s13421-016-0620-0

**Published:** 2016-05-12

**Authors:** Stephen A. Dewhurst, Rachel J. Anderson, Lydia Grace, Lotte van Esch

**Affiliations:** 1 Department of Psychology, University of Hull, Cottingham Road, Hull, HU6 7RX UK; 2 Laboratory of Experimental Psychology, University of Leuven, Leuven, Belgium

**Keywords:** False memory, Adaptive memory, Future thinking

## Abstract

Previous research has shown that rating words for their relevance to a future scenario enhances memory for those words. The current study investigated the effect of future thinking on false memory using the Deese/Roediger–McDermott (DRM) procedure. In Experiment 1, participants rated words from 6 DRM lists for relevance to a past or future event (with or without planning) or in terms of pleasantness. In a surprise recall test, levels of correct recall did not vary between the rating tasks, but the future rating conditions led to significantly higher levels of false recall than the past and pleasantness conditions did. Experiment 2 found that future rating led to higher levels of false recognition than did past and pleasantness ratings but did not affect correct recognition. The effect in false recognition was, however, eliminated when DRM items were presented in random order. Participants in Experiment 3 were presented with both DRM lists and lists of unrelated words. Future rating increased levels of false recognition for DRM lures but did not affect correct recognition for DRM or unrelated lists. The findings are discussed in terms of the view that false memories can be associated with adaptive memory functions.

In the Deese/Roediger–McDermott (DRM) procedure, named after studies by Deese (1959) and Roediger and McDermott (1995), participants study lists of words that are associates of a nonpresented critical lure (e.g., associates such as *bed*, *wake*, *rest*, and *dream* are presented but the critical lure *sleep* is not). As numerous studies have testified, this procedure gives rise to an illusion of memory in which participants erroneously claim that the critical lures were presented (see Gallo, 2010, for a review). The DRM illusion is reliably observed in tests of recall and recognition (Roediger & McDermott) and persists even when participants are forewarned about it (McDermott & Roediger, 1998). The effect has been attributed to the spontaneous activation of the critical lures in response to list items followed by source monitoring errors (Roediger, Watson, McDermott, & Gallo. 2001) or to the formation of “gist” traces that represent the theme of each list (Brainerd & Reyna, 2002).

Recently, several lines of research have led to the view that false memories, including those produced by the DRM procedure, can result from adaptive processes typically associated with positive effects on memory (see Fernandez, 2015; Howe, Garner, Charlesworth, & Knott, 2011; Schacter, 2012; Schacter, Guerin, & St. Jacques, 2011, for recent discussions of the adaptive nature of false memories). Studies that have taken an individual differences perspective have shown that ostensibly positive psychological traits can be associated with increased susceptibility to false memories. For example, Castel, McCabe, Roediger, and Heitman (2007) found that experts were at increased risk of domain-relevant intrusions when attempting to recall words related and unrelated to their domain of expertise. Castel et al. suggested that the superior organizational processes of experts support the associations that give rise to false memories. Within the DRM literature, Dewhurst, Thorley, Hammond, and Ormerod (2011) found a positive correlation between creativity, as measured by the remote associates task (RAT; Mednick, 1962), and susceptibility to false recall. In a related study, Howe, Garner, Dewhurst, and Ball (2010) found that DRM lists primed solutions to RAT problems, but only when the critical lures were falsely recalled, thereby showing that false memories can themselves be adaptive (see also Howe, Garner, Charlesworth, & Knott, 2011; Howe, Garner, & Patel, 2013; Howe, Threadgold, Norbury, Garner, & Ball, 2013). One limitation of these studies is that performance on the RAT problems involves the same associative processes that underlie the DRM illusion. More recently, however, Otgaar, Howe, van Beers, van Hoof, and Bronzwaer (2015) found that performance on a perceptual closure task (identifying degraded words) was enhanced when the targets were critical lures from previously studied DRM lists. According to Otgaar et al., the importance of this finding from an adaptive perspective is that it links false memories to performance on a task typically associated with intelligence (e.g., Luteijn & Barelds, 2004).

The idea that false memories may be functional is not new (see Johnson & Raye, 1998; Roediger, 1996, for discussions of the functions of false memories). This view has, however, gained momentum recently, and several studies have directly investigated the adaptive functions of false memories. A particularly salient illustration of the adaptive nature of false memories was provided by Howe and Derbish (2010), who investigated the effects of a survival rating task on correct and false recognition. A compelling programme of research by Nairne and colleagues (e.g., Nairne & Pandeirada, 2008; Nairne, Pandeirada, & Thompson, 2008; Nairne, Thompson, & Pandeirada, 2007) has shown that rating words for their relevance to a survival scenario, such as being stranded in the grasslands of a foreign country, leads to higher levels of correct recall and recognition than other elaborative encoding tasks do, including generation, self-reference, pleasantness ratings, and rating the words for their relevance to a nonsurvival scenario, such as moving house. Howe and Derbish replicated the survival advantage in correct recognition and, in addition, found that survival rating led to higher levels of false recognition relative to the moving house and pleasantness tasks. Howe and Derbish speculated that processing information in terms of its survival value primes related information that guides attention to other aspects of an environment that may themselves be useful for survival. In a similar study, Otgaar and Smeets (2010) found that survival rating increased correct and false recall with both DRM lists and lists of categorized words. They also showed that survival rating increased correct and false recall of DRM lists in younger and older children.

Recent research into the adaptive nature of memory, both true and false, has focused on the survival paradigm. Yet, according to Klein, Cosmides, Tooby, and Chance (2002), a more fundamental role of memory is to support the planning of future behaviour (see also Schacter, Addis, & Buckner, 2008). This view was supported in a recent study by Klein, Robertson, and Delton (2011), in which they showed that planning is the crucial element in producing the memory advantage in survival rating. They compared conditions in which participants rated words for their relevance to scenarios that involved (i) survival and planning, (ii) planning but not survival, and (iii) survival but not planning. They found higher levels of correct recall in the conditions involving planning relative to the condition that involved only survival. This future-oriented function of memory was previously demonstrated by Klein, Robertson, and Delton (2010), who instructed participants to recall a previous camping trip or to imagine a future camping trip. Participants were then presented with a list of object nouns, either related or unrelated to camping, and asked to rate the likelihood of encountering each object on the camping trip. Klein et al. found higher levels of correct recall following the future rating task relative to the past rating task. Future rating also led to higher levels of recall than survival and atemporal encoding tasks did.

The aim of the current study was to investigate the effects of future-oriented rating tasks on false memory. If, as proposed by Howe and Derbish (2010), rating DRM lists for their survival value increases false memories by activating related information that might also aid survival, then a similar increase in false memories should emerge in a future rating task via the activation of related information that might aid planning. Based on the procedure developed by Klein et al. (2010), participants imagined a past or a future scenario. They were then presented with a series of DRM items and instructed to rate them for their relevance to the imagined scenario. Participants in a control condition rated the items for pleasantness. We also attempted to isolate the effects of planning by using two versions of the past and future rating tasks; a standard version based on the procedure developed by Klein et al. and an alternative version in which planning was emphasized (see Klein et al., 2011, who found that planning mediates the effect of survival processing on correct recall). Based on the previous findings that false memories in the DRM procedure can be associated with adaptive memory processes, our prediction was that the future-oriented rating tasks would produce higher levels of false memories than the past and pleasantness rating tasks would. Experiment 1 tested this prediction by measuring free recall after each list. Experiments 2 and 3 extended the investigation to tests of recognition memory.

## Experiment 1

### Method

#### Participants

One hundred twenty undergraduates (93 females) from the University of Hull participated for course credit. All were native English speakers between the ages of 18 and 28 years (*M* = 20.12, *SD* = 2.00). Participants were tested at individual workstations in groups of up to five.

#### Stimuli and design

Six DRM lists of 10 items each were adapted from Stadler, Roediger, and McDermott (1999) and consisted of associates of the critical lures *cold*, *anger*, *doctor*, *sleep*, *sweet*, and *mountain* (presented in this order for half the participants and in the reverse order for the remaining participants). Study items were blocked by list, and items within each list were presented in descending order of backwards associative strength (strength of association from the list item to the critical lure). The five orienting tasks were past, past (planning), future, future (planning), and pleasantness. These were manipulated between participants, with 24 in each group.

#### Procedure

Participants in the pleasantness condition were told that the purpose was to investigate emotional responses to verbal stimuli. The remaining participants were told that the purpose was to investigate the processes involved in thinking about past (or future) events. An incidental learning procedure was adopted whereby no mention was made of the forthcoming memory test. Prior to the presentation of the study lists, participants received one of the following sets of instructions:

##### Past condition

*Think back and remember a specific time in your past when you went on a holiday. Try to remember specific details of this holiday*, *such as the things you did*, *the people you were with*, *the sights and sounds you experienced*, *and so on. While you are remembering this holiday*, *I am going to present you with a list of words. Using the response sheet provided*, *I would like you to rate how relevant each of these words was to the holiday you remember. Some may be relevant and others may not. It*’*s up to you to decide. Please spend a few moments remembering this holiday and wait for further instructions*.

##### Past (planning) condition

*Think back and remember a specific time in your past when you planned a holiday. Think about the preparations you had to make and the things you had to organize before you went on the holiday. While you think about the planning that was involved*, *I am going to present you with a list of words. Using the response sheet provided*, *I would like you to rate the relevance of each item to this planning. Some may be relevant and others may not. It*’*s up to you to decide. Please spend a few moments remembering the planning that went into this holiday and wait for further instructions*.

##### Future condition

*Think ahead and imagine a specific time in your future when you will go on a holiday. Try to imagine specific details of this holiday*, *such as the things you will do*, *the people you will be with*, *the sights and sounds you will experience*, *and so on. While you are imagining this holiday*, *I am going to present you with a list of words. Using the response sheet provided*, *I would like you to rate how relevant each of these words will be to the holiday you imagine. Some may be relevant and others may not. It*’*s up to you to decide. Please spend a few moments imagining this holiday and wait for further instructions*.

##### Future (planning) condition

*Imagine that you are planning a holiday. Think about the preparations you will need to make and the things you will have to do before you can go on the holiday. While you think about the planning involved*, *I am going to present you with a list of words. Using the response sheet provided*, *I would like you to rate the relevance of each item to this planning. Some may be relevant and others may not. It*’*s up to you to decide. Please spend a few moments imagining the planning that will go into this holiday and wait for further instructions*.

##### Pleasantness condition

*I am going to present you with a list of words. Using the response sheet provided*, *I would like you to rate how pleasant you think each word is. It is up to you how you define pleasantness. It can refer to the meaning of the word*, *the sound of the word*, *or some other property. It*’*s up to you to decide*.

In order to allow sufficient time for the rating tasks, study items were presented one at a time at a rate of one every 10 seconds. Each list was preceded by the list number (List 1, List 2, etc.). Participants in the past and future conditions were asked to rate the relevance of each word on a 5-point scale (1 = *totally irrelevant* to 5 = *totally relevant*). The rating scale for the pleasantness condition ranged from 1 = *very unpleasant* to 5 = *very pleasant*. After the presentation of the final list, participants were engaged in simple math problems for 2 minutes. They were then given a surprise recall test in which they were instructed to recall as many words as possible, in any order. Participants were allowed 5 minutes to complete the recall test.

#### Results and discussion

Table 1 shows mean numbers of list items correctly recalled and critical lures falsely recalled as a function of rating task. Correct and false recall data were initially analyzed in separate one-way ANOVAs, with rating task as the between-groups variable. Alpha was set at .05 in this and all other analyses. The analysis of correct recall found no reliable effect of rating task, *F* < 1. However, a significant main effect was observed in the false recall of critical lures, *F*(4, 119) = 14.07, *MSE* = .86, *p* < .001. Bonferroni-adjusted pairwise comparisons showed that, relative to the pleasantness condition, levels of false recall were significantly higher in the future and future (planning) conditions, both *p*s < .001. Levels of false recall were also higher in the past and past (planning) conditions relative to the pleasantness condition, but these differences fell short of statistical significance, both *p*s = .06. Levels of false recall were significantly higher in the future condition than the past condition and past planning conditions, both *p*s = .03. The future (planning) condition also produced significantly higher levels of false recall than the past and past (planning) conditions, both *p*s = .003. The future and future (planning) conditions did not differ significantly from each other, *p* = 1. Unrelated intrusions were low and not significantly affected by rating task, *F* < 1.

**Table 1:** Mean numbers (with standard deviations) of studied items correctly recalled (max = 60), critical lures falsely recalled (max = 6), and unrelated intrusions as a function of rating task

Study condition	List items	Critical lures	Unrelated intrusions
*Past*	27.88 (4.50)	1.33 (.87)	.58 (.77)
*Future*	26.33 (6.23)	2.17 (1.17)	.63 (.82)
*Past* (*planning*)	25.96 (6.54)	1.33 (.92)	.67 (.82)
*Future* (*planning*)	24.63 (5.35)	2.33 (.92)	.50 (.83)
*Pleasantness*	26.04 (6.80)	0.58 (.71)	.29 (.62)

The effects of temporal direction and planning were further analyzed in a 2 (past vs. future) × 2 (planning vs. no planning) between-groups ANOVA. The analysis of correct recall showed nonsignificant effects of temporal direction, *F*(1, 92) = 1.52, *p* = .22, η_p_^2^ = .02, and planning, *F*(1, 92) = 2.42, *p* = .12, η_p_^2^ = .03, and a nonsignificant interaction, *F* < 1. In contrast, the analysis of false recall showed a significant effect of temporal direction, *F*(1, 92) = 21.24, *p* < .001, η_p_^2^ =.19, whereby future rating led to higher levels of false recall than past rating. Neither the effect of planning nor the interaction were significant, both *F*s < 1.

The ratings provided during the study phase were analyzed in a one-way ANOVA. The main effect of rating task was significant, *F*(4, 119) = 9.37, *MSE* = .19, *p* < .001. Bonferroni-adjusted pairwise comparisons showed that mean ratings in the pleasantness condition (2.80) were significantly higher than ratings in the past (2.26), future (2.35), past (planning) (2.15) and future (planning) (2.33) conditions, all *p*s<.005, which did not differ significantly from each other.

The main finding from Experiment 1 is that rating DRM lists for their relevance to a future event leads to reliably higher levels of false recall than rating them for their relevance to a past event. This was the case irrespective of whether the rating tasks involved planning or not. Adding a planning component to the past and future conditions did not significantly increase levels of false recall. In contrast to the findings of Klein et al. (2010), the future rating tasks did not lead to higher levels of correct recall than the past rating tasks did. In fact, no significant differences in correct recall were observed between any of the rating tasks. A possible explanation for the null effects in correct recall is that any potential effects of rating task were overshadowed by the semantic theme of the DRM lists. This was investigated in Experiment 2, in which we manipulated the organization of the DRM lists.

## Experiment 2

The primary aim of Experiment 2 was to determine whether the effects of future thinking on false recall extend to false recognition. Because there were no significant differences between the planning and no planning conditions, this manipulation was dropped from Experiment 2, and only the future (planning), past (planning), and pleasantness conditions were retained. The three rating tasks were manipulated within groups. As noted previously, a further aim was to investigate the possibility that the null effect of temporal direction in correct memory was due to the associative nature of the DRM lists. To this end, Experiment 2 incorporated a between-groups manipulation of list structure whereby, for half the participants, the DRM lists were presented in a blocked list-by-list sequence, and for the remaining participants the words were presented in a randomized order. Toglia, Neuschatz, and Goodwin (1999) found that blocked presentation reduced the magnitude of the DRM illusion, a finding they attributed to a reduction in the saliency of the semantic themes of the individual lists. If the null effect of future thinking in correct memory observed in Experiment 1 was due to the saliency of the list themes, reducing this saliency should allow effects of future thinking to emerge in correct memory.

### Method

#### Participants

A new group of 80 undergraduate students (49 females) from the University of Hull participated for course credit. They were native English speakers in the age range 18 to 57 years (*M* = 21.86, *SD* = 7.74). They were tested at individual workstations in groups of up to five.

#### Stimuli and design

Each participant rated 24 DRM lists of 10 items each, with eight lists in each rating condition. For 40 of the participants, the 80 words within each condition were presented in a blocked, list-by-list sequence, with the words in each list presented in order of backwards associative strength. For the remaining 40 participants, the 80 words within each condition were presented in a random order. Allocation of list to rating condition and the orders in which conditions and lists were presented were rotated across participants. The recognition test consisted of three words from each list (the critical lure plus studied items from positions 3 and 5). The corresponding items from eight unstudied DRM lists provided the unrelated distractors in the recognition test.

#### Procedure

The rating instructions were the same as those used in Experiment 1. Participants read the first set of instructions and then rated eight DRM lists accordingly. The words were presented at a rate of one every 5 seconds and responses were recorded using ePrime software. Each word remained on the screen for the full 5 seconds regardless of response time. This procedure was repeated for the remaining two rating tasks. After the final list had been rated, participants were engaged in simple math problems for 10 minutes. The surprise recognition test was then administered using ePrime software.

#### Results and discussion

Table 2 shows mean proportions of hits and false alarms as a function of list structure and rating task. These were analyzed in separate 2 (structure: blocked vs. random) × 3 (rating task: past vs. future vs. pleasantness) ANOVAs with repeated measures on the second factor. Analysis of correct recognition showed a significant main effect of structure, *F*(1, 78) = 5.69, *MSE* = 23.20, *p* = .019, η_p_^2^ = .07. As can be seen from Table 2, hit rates were higher following blocked presentation rather than random presentation. Neither the main effect of rating task nor the interaction with structure were significant, *F* < 1. The analysis of false alarms to critical lures showed nonsignificant main effects of structure, *F* < 1, and rating task, *F* < 1.3. There was, however, a significant interaction between structure and rating task, *F*(2, 156) = 6.68, *MSE* = 2.06, *p* = .002, η_p_^2^ = .08. Bonferroni-adjusted pairwise comparisons showed that, in the blocked condition, future rating led to higher levels of false recognition of critical lures than both past rating, *p* = .041, and pleasantness rating, *p* = .006, which did not differ reliably from each other, *p* = 63. There were no significant pairwise comparisons in the random structure condition. The mean proportions of unrelated lures falsely recognized were .13 in the blocked condition and .17 in the random condition.

**Table 2:** Mean proportions (with standard deviations) of studied items correctly recognized and critical lures falsely recognized as a function of structure and rating task

List structure	Studied items	Critical lures
*Blocked*		
*Past* (*planning*)	.84 (.14)	.56 (.20)
*Future* (*planning*)	.87 (.12)	.66 (.20)
*Pleasantness*	.86 (.12)	.51 (.21)
*Random presentation*		
*Past* (*planning*)	.76 (.22)	.54 (.28)
*Future* (*planning*)	.75 (.27)	.51 (.29)
*Pleasantness*	.78 (.24)	.58 (.28)

Study ratings were also analyzed in a 2 (structure) × 3 (rating task) ANOVA. This yielded a significant main effect of structure, *F*(1, 78) = 4.18, *MSE* = .66, *p* = .044, η_p_^2^ = .05, whereby ratings were higher overall in the random condition (*M* = 2.59) than in the blocked condition (*M* = 2.37). There was also a significant main effect of rating task, *F*(2, 156) = 44.64, *MSE* = .19, *p* < .001, η_p_^2^ = .36. Bonferroni-adjusted pairwise comparisons showed that mean ratings in the pleasantness condition (2.86) were significantly higher than in both the past (2.30) and future (2.28) conditions, both *p*s < .001, which did not differ significantly from each other, *p* = 1. The interaction between structure and rating task was not significant, *F* < 1.

The results of Experiment 2 extend the effects of future rating on false memory from free recall to recognition. Specifically, the future rating condition led to higher levels of false recognition than the past rating and pleasantness rating conditions. The results of Experiment 2 also confirm that the effect of future rating persists when the rating tasks are manipulated in a within-subjects design. A caveat to this, however, is that the effect of future rating on false recognition was only observed when the DRM lists were presented in a blocked design. Presenting the study items in a random order eliminated the effect. This contrasts with the findings of Howe and Derbish (2010) that survival rating increased false memory when DRM lists were presented in random sequences. This is considered further in the general discussion.

As in Experiment 1, rating task did not reliably influence levels of true memory. These null effects are in contrast to the findings of Klein et al. (2010), that rating words for their relevance to a future camping trip produced higher levels of recall than rating the words for their relevance to a past camping trip. As discussed previously, it is possible that the null effects of temporal direction observed in the current study were due to the use of DRM lists, in which the studied items are highly associated with each other as well as with the critical lure. Although random presentation eliminated the effects of future thinking on false recognition, levels of false recognition overall were still relatively high (see Table 2), which suggests that random presentation did not prevent participants from making associations at study. In order to investigate this further, we conducted Experiment 3 in which participants studied both DRM lists and lists of unrelated nouns.^1^ As in Experiment 2, participants rated the words for relevance to a past event, a future event, or in terms of pleasantness, in a within-subjects design. If the null effects in true memory observed in Experiments 1 and 2 were due to the use of DRM lists, then an effect of future thinking should occur in the unrelated lists but not in the DRM lists.

## Experiment 3

### Method

The method was the same as Experiment 2 with the following modifications: A new group of 45 participants (33 females) were recruited. All were native English speakers between the ages of 18 and 23 years (*M* = 19.44, *SD* = 1.25). Participants studied six DRM lists in each of three conditions: relevance to a future holiday, relevance to a past holiday, and pleasantness. In addition to the six DRM lists, participants rated six lists of 10 unrelated common nouns in each condition, with DRM and unrelated lists alternating. The words were selected from the MRC Psycholinguistic Database (Coltheart, 1981). Four sets of DRM and unrelated lists were created and rotated through the three rating conditions. The fourth set provided the unrelated distractors in the recognition test. The recognition test consisted of 64 studied items (two from each list), the 18 critical lures from DRM lists, 18 distractors from six unstudied DRM lists, and 18 distractors from unstudied unrelated lists.

### Results and discussion

Table 3 shows mean levels of correct recognition for the DRM and unrelated lists and mean levels of false recognition for DRM lists. Correct recognition scores were analyzed in a 2 (list type: DRM vs. unrelated) × 3 (rating task: past vs. future vs. pleasantness) repeated-measures ANOVA. A significant main effect of list type was observed whereby correct recognition scores were higher for unrelated lists than for DRM lists, *F*(1, 44) = 7.01, *MSE* = 1.60, *p* = .011, η_p_^2^ = .14. A significant main effect of rating task was also observed, *F*(2, 88) = 11.13, *MSE* = 1.98, *p* = .001, η_p_^2^ = .20. Bonferroni-adjusted pairwise comparisons showed that pleasantness ratings led to significantly higher levels of correct recognition than both future rating, *p* = .046, and past rating, *p* < .001. The interaction between list type and rating task was not significant, *F*(2, 88) = 1.81, *MSE* = 2.21, *p* = .17, η_p_^2^ = .04. A one-way ANOVA conducted on the critical lures of DRM lists showed a significant main effect of rating task, *F*(2, 88) = 5.33, *MSE* = 1.10, *p* = .007, η_p_^2^ = .11. Bonferroni-adjusted pairwise comparisons showed that future rating led to higher levels of false recognition of critical lures than did past rating, *p* = .006, and pleasantness rating, *p* = .044, which did not differ from one another, *p* = 1. The mean proportion of unrelated lures falsely recognized was .17.

**Table 3:** Mean proportions (with standard deviations) of studied DRM and studied unrelated items correctly recognized and critical lures falsely recognized as a function of rating task

Study condition	DRM items	Critical lures	Unrelated items
*Past* (*planning*)	.63 (.18)	.37 (.19)	.70 (.15)
*Future* (*planning*)	.69 (.17)	.47 (.22)	.71 (.15)
*Pleasantness*	.74 (.09)	.37 (.22)	.75 (.10)

Study ratings were analyzed in a 2 (list type: DRM vs, unrelated) × 3 (rating task: past vs. future vs. pleasantness) repeated-measures ANOVA. The main effect of list type was not significant, *F* < 1. There was, however, a significant main effect of rating task, *F*(2, 88) = 53.23, *MSE* = .33, *p* < .001, η_p_^2^ = .55. Bonferroni-adjusted pairwise comparisons showed that mean ratings were significantly higher for the pleasantness condition (2.91) than for the past (2.11) and future (2.20) conditions, both *p*s < .001, which did not differ reliably from each other, *p* = .39. The interaction between list type and rating was not significant, *F*(2, 88) = 1.39, *MSE* = .03, *p* = .25, η_p_^2^ = .03.

The findings of Experiment 3 confirm those of Experiments 1 and 2 in showing that rating words in relation to a future event leads to higher levels of false memory than rating them in relation to a past event or in terms of pleasantness. The main objective of Experiment 3 was to investigate whether the null effects of future thinking in true memory were due to the use of DRM lists. As in Experiments 1 and 2, future thinking did not increase levels of correct recognition of DRM lists, relative to past thinking or pleasantness. Nor did future thinking increase correct recognition of unrelated lists. In fact, correct recognition scores were significantly lower in the past and future conditions relative to the pleasantness condition. These findings suggest that the null effects of future thinking in true memory are not due to the use of DRM lists. This issue is considered further in the general discussion. The finding that correct recognition scores were higher for unrelated lists than for DRM lists is likely due to the differences between the lists in terms of grammatical class. The unrelated lists consisted of common nouns similar to those typically used in the survival paradigm (e.g., Nairne et al., 2007). In contrast, DRM lists include adjectives, verbs, and abstract nouns, which are less memorable than common nouns (e.g., Paivio, 1971; Simpson & Klippert, 1968).

## General discussion

The main finding from the current study is that rating DRM lists for their relevance to a future scenario led to higher levels of false recall than rating them for their relevance to a past scenario or in terms of pleasantness. This effect was observed in both free recall (Experiment 1) and recognition memory (Experiments 2 and 3). These findings add to the growing body of evidence that false memories can result from adaptive processes that usually have positive consequences for memory. Previous studies have shown that false memories can prime problem solutions (e.g., Howe et al., 2010; Otgaar et al., 2015) and are increased by survival rating (Howe & Derbish, 2010; Otgaar & Smeets, 2010). The current study extends these findings by showing that similar increases in false memory are produced by future thinking. The effects of temporal direction cannot be attributed to differences in the study ratings, because the only reliable differences to emerge were significantly higher ratings for pleasantness relative to the other rating tasks.

In Experiment 1, half the participants in the past and future conditions were explicitly instructed to think about the planning involved in the holiday they remembered or imagined. Although the future (planning) condition led to the highest levels of false recall numerically, the emphasis on planning did not significantly increase levels of false recall. In contrast, Klein et al. (2011) found that adding a planning component to a survival processing paradigm led to higher levels of correct recall than standard survival instructions. In an earlier study, Klein et al. (2010) showed that the effect of survival processing is mediated by the future-orientation of the survival condition, rather than survival per se, which they attributed to an implicit planning requirement. Although Klein and colleagues did not measure false recall, their findings, along with those of Experiment 1, suggest that planning is inherently involved in future-oriented tasks. Adding an explicit planning requirement does not significantly increase the levels of correct or false recall beyond those produced by future thinking.

Although the future rating tasks led to higher levels of false memory than the past and pleasantness rating tasks did, we did not replicate the finding by Klein et al. (2010, 2011) of higher levels of true memory in the future conditions. The divergent results are likely to reflect procedural differences. The most salient difference is that we used DRM lists whereas Klein et al. used schema-related stimuli (e.g., words related to a camping scenario). It is possible that the semantic themes of the DRM lists overshadowed the effect of rating task and led to equivalent levels of correct recall and recognition in all conditions. A problem with this explanation is that the manipulation of rating task did not reliably affect correct recognition in Experiment 2 when items from DRM lists were presented in random sequences, or in Experiment 3 when participants studied unrelated lists. However, as noted in the discussion of Experiment 2, random presentation of DRM stimuli does not eliminate the semantic themes of the lists, it merely reduces their salience. It is likely that the associative nature of DRM stimuli overshadowed the effects of rating task even with random presentation. Regarding Experiment 3, it is possible that the presentation of DRM lists at study influenced the encoding and retrieval of unrelated lists, though the precise mechanism for this is unclear. We are currently investigating whether the presentation of schema-related stimuli, similar to those used by Klein et al., can lead to effects of future thinking in both correct and false memory.

There are other important differences between the current study and those reported by Klein et al. (2010, 2011). For example, Klein et al. used a between-subjects manipulation of rating task followed by tests of free recall. In contrast, Experiments 2 and 3 of the current study used a within-subjects manipulation of rating task followed by tests of recognition memory. In addition, Klein et al. instructed participants to remember or imagine a camping trip, whereas participants in the present study remembered or imagined a holiday. Identifying the boundary conditions of the effects reported by Klein and colleagues is beyond the scope of the current study. It is important to note, however, that we observed null effects in correct memory in three experiments, in tests of both recall and recognition, and following both between- and within-subjects manipulations of rating task. Although the differential effects in correct memory have yet to be explained, our focus in the current study was on the effects on future rating on false memory. We have demonstrated a consistent and replicable pattern whereby rating words for their relevance to a future event leads to higher levels of false memory than rating them for their relevance to a past event.

Although the null effect of future thinking on correct memory is at odds with the findings of Klein et al. (2010, 2011), the overall pattern of results is consistent with data reported by Nairne et al. (2007). Nairne et al. (Experiment 1) compared the effects on free recall of rating words for their relevance to survival, to moving house, or for pleasantness. From the perspective of the current study, the most salient finding is that, relative to pleasantness ratings, the moving scenario (which could be interpreted as a future planning task) had no effect of correct recall but led to significantly higher levels of false recall.^2^ Although false alarm rates were low (the stimuli were unrelated nouns rather than DRM lists), the fact that Nairne et al. observed the same dissociation between correct and false memory as observed in the current study provides indirect support for the validity of the current findings. The pattern is also consistent with findings reported by Dewhurst, Bould, Knott, and Thorley (2009), who found that explicitly instructing participants to make associations to studied DRM items increased false recognition but had no effect on correct recognition. The current findings suggest that merely facilitating the generation of associates can elicit the same pattern.

The activation-monitoring account of the DRM illusion (Roediger et al., 2001) can explain the current findings if one assumes that imagining a future event facilitates associative processes at encoding, relative to remembering a past event. Support for this possibility comes from research showing that imagining future events leads to more flexible thinking than does remembering past events (see D’Argembeau, Ortoleva, Jumentier, & van der Linden, 2010). As discussed by Anderson, Dewhurst, and Nash (2012), it may be adaptive to represent future events in a less specific and more schematized form than past events. In terms of the current study, the greater flexibility of future thinking may have enabled participants to think creatively about the possibility of encountering studied items in a hypothetical future event, thereby increasing the possibility of activating the critical lure. In terms of fuzzy-trace theory (Brainerd & Reyna, 2002), it is possible that future thinking facilitates the formation of gist traces, which give rise to false memories, but has no effect on verbatim traces, which support correct memories.

The current findings do not, therefore, arbitrate between activation-monitoring theory and fuzzy-trace theory. Nevertheless, the increase in false but not correct memory following future rating is particular salient for theories of the DRM illusion, as this pattern has rarely been observed in previous research. The only example we are aware of is the finding by Dewhurst et al. (2009), that explicitly instructing participants to make associations to studied items increased false but not correct recognition. Another recent study from our lab (Dewhurst, Rackie, & van Esch, 2016) found precisely the opposite pattern whereby a manipulated variable increased correct but not false memory. Specifically, we found that translating between modalities at study (e.g., writing a word presented auditorily) increased correct recognition but had no effect on false recognition. This is analogous to the “generation at no cost” pattern reported by Soraci, Carlin, Toglia, Chechile, and Neuschatz (2003). They found that generating DRM study items from fragments increased correct recall and recognition but had no reliable effect on false recall or recognition. In contrast, numerous studies have reported a “more is less” pattern, whereby an independent variable simultaneously increases correct and false memory (Toglia et al., 1999). Less common in the DRM literature is the finding of an independent variable that simultaneously increases correct memory and reduces false memory (see Benjamin, 2001, for an example). Any theory of memory must be able to account for these different patterns (see Dewhurst et al. 2015, for further discussion of this). The more examples we can find of the less common permutations, such as the pattern observed in the current study, the easier it will be to identify the processes that underlie the DRM illusion.

The current findings can also be contrasted with those of Howe and Derbish (2010), who found that survival rating increased both correct and false memory using DRM lists. Howe and Derbish presented DRM lists in random sequences, but Otgaar and Smeets (2010) found that these effects also occur with blocked presentation. These studies found parallel effects in true and false memory, whereas the current study found effects only in false memory. It is important to note, however, that survival rating and future planning are not the same task. Although the two tasks share some commonalities (e.g., both require participants to imagine a hypothetical scenario), the present findings suggest important differences in terms of their effects on memory. For example, Klein et al. (2011) found that the effect of survival rating on correct recall was enhanced when instructions emphasized planning. In contrast, the addition of a planning component in Experiment 1 of the current study did not enhance the effect of future thinking on false recall. A useful goal for future research would be to attempt to disentangle the effects of survival rating, planning, and future thinking on correct and false memory.

In conclusion, the current findings lend further support to the emerging view that false memories and memory illusions can result from mnemonic processes that typically have a positive influence on memory (Howe, 2011; Schacter et al., 2011). Previous research has shown that rating DRM lists for their relevance to a survival situation increases false memories of the critical lures (Howe & Derbish, 2010; Otgaar & Smeets, 2010). The current study extends this pattern to a planning task by showing that rating words for their relevance to a future scenario leads to higher levels of false recall and false recognition than rating their relevance to a past scenario or rating them in terms of pleasantness. There are, however, a number of differences between the effects of survival rating and the effects of future thinking on correct and false memory. Despite these, the current findings are consistent with the views of Klein et al. (2002) that one of the primary functions of human memory is to support the planning of future acts. The current findings illustrate the generality of this future-oriented function of memory by showing that the associative processes that give rise to false memories are also enhanced by future planning.

## Footnotes

1 We thank Henry Otgaar for this suggestion.

2 We are grateful to Jim Nairne for drawing our attention to this.
